# Improved Compressed Sensing-Based Algorithm for Sparse-View CT Image Reconstruction

**DOI:** 10.1155/2013/185750

**Published:** 2013-03-31

**Authors:** Zangen Zhu, Khan Wahid, Paul Babyn, David Cooper, Isaac Pratt, Yasmin Carter

**Affiliations:** ^1^Department of Electrical and Computer Engineering, University of Saskatchewan, Saskatoon, Canada S7N 5A9; ^2^Department of Medical Imaging, Saskatoon Health Region, Saskatoon, Canada S7N 0W8; ^3^College of Medicine, University of Saskatchewan, Saskatoon, Canada S7N 5E5

## Abstract

In computed tomography (CT), there are many situations where reconstruction has to be performed with sparse-view data. In sparse-view CT imaging, strong streak artifacts may appear in conventionally reconstructed images due to limited sampling rate that compromises image quality. Compressed sensing (CS) algorithm has shown potential to accurately recover images from highly undersampled data. In the past few years, total-variation-(TV-) based compressed sensing algorithms have been proposed to suppress the streak artifact in CT image reconstruction. In this paper, we propose an efficient compressed sensing-based algorithm for CT image reconstruction from few-view data where we simultaneously minimize three parameters: the *ℓ*_1_ norm, total variation, and a least squares measure. The main feature of our algorithm is the use of two sparsity transforms—discrete wavelet transform and discrete gradient transform. Experiments have been conducted using simulated phantoms and clinical data to evaluate the performance of the proposed algorithm. The results using the proposed scheme show much smaller streaking artifacts and reconstruction errors than other conventional methods.

## 1. Introduction

X-ray computed tomography (CT) is extensively used clinically to evaluate patients with a variety of conditions. However, by its nature, CT scans expose the patients to high X-ray radiation doses which can result in an increased lifetime risk of cancer [1, 2]. The radiation dose to the patients is proportional to the number of X-ray projections. Additionally, medical research makes extensive use of CT on the microscopic scale, known as micro-CT. Longitudinal studies on experimental animals such as rats, mice, and rabbits are also restricted in resolution and image quality by radiation dose. Currently, the defacto standard for reconstruction on the commercial CT scanners is the filtered backprojection (FBP) algorithm, which typically requires a large number (300–1000) of angular views for yielding accurate reconstruction of the image object.

Recently a number of strategies have been proposed to decrease radiation dose in CT scans. One approach to lower the total X-ray radiation dose is to simply reduce the dose level mAs/view in data acquisition protocols. This approach typically results in an insufficient number of X-ray photons received by the detectors, increasing the noise level on the sinograms produced. The noise-contaminated sinogram data will degrade the quality of reconstructed CT images when a conventional FBP algorithm is used [3]. Another way to reduce the total radiation dose is to reduce the number of projections needed. According to the standard image reconstruction theory in image processing, when the number of the view angles does not satisfy the Shannon/Nyquist sampling theorem, aliasing artifacts will spread out in the reconstructed images. As a consequence, FBP algorithms do not produce diagnostically satisfactory images in sparse-view data collection schemes, because they are derived by assuming densely sampled projections over the scanning angular range. 

Since analytical reconstruction methods, such as FBP, cause such serious streaking artifacts in the resulting reconstructed CT images, iterative algorithms have been proposed and investigated as a means to eliminate these defects. One approach is algebraic and is based upon solving a system of linear equations. This scheme is often referred to as algebraic reconstruction technique (ART) [4, 5], and it has several variants with different iteration schemes, such as simultaneous ART (SART) [6, 7]. The ART algorithms consist of altering the grayness of each pixel intersected by the ray sum in such a way as to make the ray sum agree with the corresponding element of the measured projection. In each iteration, the current guess of the image is reprojected and checked to see how it matches with the real measurements. These algebraic methods are computationally intensive and require large amounts of memory [8]. However increases in computing power may render them more available over time. Other iterative approaches, such as statistical image reconstruction (SIR) [9], use the statistical distribution of photons resulting from the X-ray interaction process. Both the ART and SIR methods solve the reconstruction problem iteratively. Iterative algorithms have been proven to be advantageous over analytical algorithms when projection data are incomplete and noisy, for example, in the sparse-view reconstruction scenario. However, when the Shannon/Nyquist sampling requirement is violated, that is, less than 100 view angles, the linear system will become highly underdetermined and unstable, failing to maintain clinically acceptable image quality. 

In the past few years, compressed sensing (CS) algorithms [10, 11] have attracted huge attention in the CT and micro-CT community. One may view CS-based algorithm as simply another iterative algorithm, but what makes the CS method distinctive from other iterative algorithms is that it exploits the sampling strategy in which the sampled data are truly helpful for an accurate reconstruction of an image object. Several compressed sensing based CT image reconstruction algorithms are proposed in the sparse-view scenario [12, 13]. In particular, the total-variation-(TV-) based methods have demonstrated their power in CT reconstruction with only a few X-ray projections with their dataset. For example, Sidky's work in [13] showed that their method can yield accurate reconstructions in ideal conditions where only 20 view angles projection data were acquired using simulated data from a jaw phantom. In such algorithms, an objective function of TV norm is minimized subject to a data fidelity posed by the acquired projection data. Minimizing the image gradient essentially suppresses those high spatial frequency parts such as streaking artifacts and noise in the reconstructed images. The major problem of this TV-based compressed sensing method is that it tries to uniformly penalize the image's gradient irrespective of the underlying image structures and thus low contrast regions are sometimes over smoothed [3]. To resolve this issue, we propose a new algorithm based on compressed sensing that jointly minimizes the wavelet transform and total variation of the object image. The 2D wavelet transform is good at capturing point singularities [14], thus preserving edges and low contrast information. This process suppresses the streaking artifacts and noise, while detailed structures are also preserved, resulting in an improved image. 

## 2. Theory and Method

### 2.1. X-Ray Computed Tomography (CT) Imaging System

A parallel beam CT scanning system uses an array of equally spaced sources of X-ray beams and an array of detectors. Let *μ*(*x*, *y*) denote the X-ray attenuation coefficient distribution of tissue of a 2D target object and let *l* denote the straight line from the X-ray focal spot to the detector pixel, which is also referred to as the X-ray path. The X-ray tube emits X-ray photons which travel in a straight line through the object. The photons are attenuated by the materials in the target object. Radiation that is not absorbed by the object's internal structure reaches the detectors. According to Beer's law, the detected photon number *I* and the entering photon number *I*_0_ at a given detector pixel have the following relationship [15]:

(1) $$\begin{array}{c} I=I_{0}\exp\left(-\int_{l}^{}\mu \left(x,y\right)dl\right), \end{array}$$

where the line integral is performed along the X-ray path. Alternatively, one can define

(2) $$\begin{array}{c} y=\int_{l}^{}\mu \left(x,y\right)dl=\ln\frac{I_{0}}{I}, \end{array}$$

where *y* is the so-called projection data or sinogram, which is essentially the line integral in (1). Then the image reconstruction process consists of estimating the attenuation coefficients, *μ*, from the detected projection data *y*. In computer implementation, the attenuation coefficients are digitized into the so-called pixel representations [16]:

(3) $$\begin{array}{c} \mu \left(x,y\right)=\sum_{i\in S}\mu_{i}\omega_{i}\left(x,y\right), \end{array}$$

where *S* denotes the index of the set of *N* pixel locations, *i* is the pixel index, and *ω*_*i*_(*x*, *y*) is the basis function. Substituting (3) into the line integral equation in (2), one can obtain

(4) $$\begin{array}{c} y=\sum_{i\in S}\mu_{i}\int_{l}\omega_{i}\left(x,y\right)dl=\sum_{i\in S}A_{ji}\mu_{i}=A\mu , \end{array}$$

where the X-ray system matrix *A* is given by

(5) $$\begin{array}{c} A_{ji}=\int_{l_{j}}\omega_{i}\left(x,y\right)dl, \end{array}$$

which is the line integral of the basis function *ω*_*i*_(*x*, *y*) along the *j*th X-ray path. The system matrix is independent of the image object; it is rather dependent on the CT scanner, including the positions of sources and detectors. Hence (4) gives a system of linear equations with *μ*_*i*_.

### 2.2. Brief Overview of Existing Methods

In this study, our proposed algorithm will be compared with the state-of-the-art methods, including filtered backprojection (FBP) [15], algebraic reconstruction technique (ART) [4, 5], and its variants, simultaneous algebraic reconstruction technique (SART) [8]. A brief summary of these methods is given in the following.

#### 2.2.1. Filtered Backprojection (FBP)

**Figure 1:**
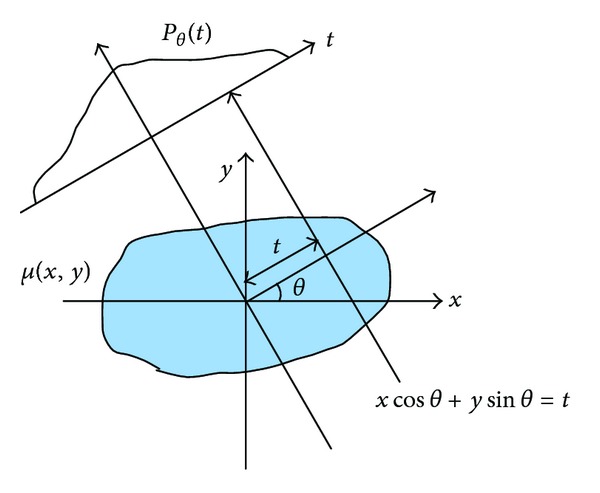
A parallel beam projection through *μ*(*x*, *y*) at angle  *θ*. *P*_*θ*_(*t*) is the measured projection.

Consider the parallel beam of rays intersecting an object as shown in Figure 1. The parallel beam is inclined to the X-axis at angle *θ* and each ray can be characterized by its perpendicular distance, *t*, to the origin. Equation (2) can be rewritten as

(6) $$\begin{array}{c} P_{\theta }\left(t\right)=\int_{l_{\theta ,t}}\mu \left(x,y\right)dl=\ln\frac{I_{0}}{I}. \end{array}$$

Using a Dirac delta function, we have an alternate representation:

(7) $$\begin{array}{c} P_{\theta }\left(t\right)=\int_{-\infty }^{\infty }\int_{-\infty }^{\infty }\mu \left(x,y\right)\delta \left(x\cos\theta +y\sin\theta -t\right)dx\;dy. \end{array}$$

FBP begins by filtering the projection data with a high pass filter, which in reality is implemented by the Ram-Lak filter or Shepp-Logan filter, then takes the integral over 0 to *π* with respect to *θ*. Since filtering in frequency domain can be done by the convolution operation in spatial domain, the formulation of filtered backprojection is

(8) $$\begin{array}{c} \mu \left(x,y\right)=\int_{0}^{\pi }d\theta \int_{-\infty }^{\infty }P_{\theta }\left(t^{\prime }\right)\varphi \left(t-t^{\prime }\right)dt^{\prime }, \end{array}$$

where *φ*(*t*) is the corresponding high pass filter in spatial domain.

#### 2.2.2. Algebraic Reconstruction Technique (ART)

ART considers the CT imaging process as a linear system of equations as in (4):

(9) $$\begin{array}{c} y=A\mu , \end{array}$$

where *A* is the system matrix (given in (5)) describing the forward projection in the CT scan. ART algorithms solve the above equations in an iterative way so that the difference between the projection data from real scan and the projection data calculated from the estimated image is backprojected onto the estimated image at current iteration step. Given that the system matrix *A* is of size *m* × *n*, the method involves the *i*th row of *A* in the following update of iteration:

(10) $$\begin{array}{c} x^{k+1}=x^{k}+\lambda_{k}\frac{b_{i}-\langle a_{i},x^{k}\rangle }{{||a_{i}||}^{2}}a_{i}, \end{array}$$

where *i* = *k* mod *m* + 1, *a*_*i*_ is the *i*th row of the matrix *A*, *b*_*i*_ is the *i*th component of the vector *b*, and *λ*_*k*_ is a relaxation parameter. In the original work in [17], Kaczmarz used a fixed *λ*_*k*_ = *λ* = 1 ∈ (0,2) and the *k*th iteration consists of a “sweep” through the *m* rows of *A*, that is, *i* = 1,2,…, *m*. Kaczmarz's method was employed in this study as comparison with the proposed algorithm. 

#### 2.2.3. Simultaneous Algebraic Reconstruction Technique (SART)

The reason for calling the methods “simultaneous” is that all the equations are used at the same time in one iteration. The general form of simultaneous iterative reconstruction technique (SIRT) is

(11) $$\begin{array}{c} x^{k+1}=x^{k}+\lambda_{k}TA^{T}M\left(b-Ax^{k}\right),\;k=0,1,2,\ldots , \end{array}$$

where the matrices *M* and *T* are symmetric positive definite. Although SART was originally developed in the framework of ART [6], it can also be written and implemented in the SIRT form and takes the following matrix form [18]:

(12) $$\begin{array}{c} x^{k+1}=x^{k}+\lambda_{k}D_{r}^{-1}A^{T}D_{c}^{-1}\left(b-Ax^{k}\right), \end{array}$$

where the diagonal matrices *D*_*r*_ and *D*_*c*_ are defined in terms of the row and column sum:

(13) $$\begin{array}{c} D_{r}=\mathrm{diag}\left({||a_{i}||}_{1}\right),\;\;D_{c}=\mathrm{diag}\left({||a^{j}||}_{1}\right). \end{array}$$

ART-type methods are known to have better performance than FBP algorithms in suppressing streak artifacts and noise in sparse-view CT imaging. 

## 3. Proposed CS-Based Algorithm

The problem of sparse-view CT image reconstruction actually leads to an underdetermined system of linear equations (equation (9)). One way to improve performance is to incorporate a priori knowledge into the iteration process. One way to do that is based on the idea of sparsity at compressed sensing [10, 11]. The essence of compressed sensing is that a signal, which in our case is the image *μ*, can be completely reconstructed with a high probability with far less samples than required by conventional Nyquist-Shannon sampling theorem, if the image has a sparse/compressible representation in a transform domain Φ, such that most entries of the vector Φ*μ* are zero or close to zero. The entire process of compressed sensing consists of three steps [19]: encoding, sensing, and decoding. In the first step, the object image *μ* of size *n* is encoded into a smaller vector *y* = *Aμ* of a size *m* (*m* < *n*) by the system matrix, as shown in Section 2.1. Then the second step is obtaining the undersampled measurements *y* from the imaging system, which in CT is to obtain the undersampled projection data. Incorporating the a priori knowledge into the process of image reconstruction, the third step is to solve the following constrained optimization problem:

(14) $$\begin{array}{c} \underset{\mu }{\min}{||\Phi \mu ||}_{1}\;\;\text{subject}\;\text{to}\;{||A\mu -y||}_{2}<\varepsilon , \end{array}$$

where *ε* is a parameter controlling the data consistency. It has been mathematically proven that, if the image has only *k* entries with relatively large magnitudes, the order of $k\ln\sqrt{n}$ measurements is sufficient to accurately reconstruct *μ* via *ℓ*_1_ norm minimization procedure with high probability (Algorithm 1). A previous method called PICCS used total variation (TV) as a sparsity transform [12], where the CT image is reconstructed by minimizing the energy function with a TV regularization term:

(15) $$\begin{array}{c} \mu =\arg\underset{\mu }{\min}J\left(\mu \right)=\arg\underset{\mu }{\min}\lambda {||\mu ||}_{\text{TV}}+{||A\mu -y||}_{2}^{2}, \end{array}$$

where the regularization factor *λ* is introduced to leverage the cost function's emphasis on the sparseness prior and the data fidelity term. The selection of this regularization factor has been an interesting area of research in the field of regularized iterative methods [20–22]. A well-known method to find the best one is via the *L* curve. In our study, we chose the optimized regularization parameter for TV method for each dataset. The discussion of selection is given in Section 4. The TV term of an image in this work is defined as follows:

(16) $$\begin{array}{c} {||\mu ||}_{\text{TV}}=\int \left|\nabla \mu \right|dx. \end{array}$$

In a discrete version, (16) becomes

(17) $$\begin{array}{c} {||\mu ||}_{\text{TV}}=\sum_{i,j}\sqrt{{\left(\nabla \mu_{x}^{2}\right)}_{i,j}+{\left(\nabla \mu_{y}^{2}\right)}_{i,j}}, \end{array}$$

where ∇*μ*_*x*_, ∇*μ*_*y*_ represent the finite differences of the image along *x* and *y* directions. Despite the great success of the TV model in terms of reconstructing high-quality images, edges with low contrast regions are sometimes oversmoothed, causing loss of low contrast information. To overcome this disadvantage, we propose a novel compressed sensing-based method by combining two sparsity transforms: TV and wavelet. Wavelet is good at preserving edges and low contrast information while TV is efficient at suppressing noise and streaking artifacts. In this way, we obtain a good balance between streaking artifacts suppression and detail preservation. Our iterative reconstruction algorithm solves the image via the following optimization problem:

(18) $$\begin{array}{c} \mu =\arg\underset{\mu }{\min}\lambda_{1}{||\mu ||}_{\text{TV}}+\lambda_{2}{||\Phi \mu ||}_{1}+{||A\mu -y||}_{2}^{2}. \end{array}$$

The two regularization factors *λ*_1_ and *λ*_2_ control the amount of smoothing. A large *λ*_1_ and small *λ*_2_ are not able to capture enough detail information. In such a circumstance, the algorithm becomes essentially the TV method. In contrast, small *λ*_1_ and large *λ*_2_ tend to give low weights to image gradients, making the method inefficient at suppressing noise and streaking artifacts. The process to find the optimized selections of *λ*_1_ and *λ*_2_ is discussed in Section 4. We exploit a fast implementation of the wavelet transform [23], which speeds up the implementation.

Since (18) poses an unconstrained convex optimization problem, we propose solving it using a nonlinear conjugate gradient descent algorithm with backtracking line search where *J*(*μ*) is the cost function as defined in (18). 

The conjugate gradient requires the computation of ∇*J*(*μ*) which is

(19) $$\begin{array}{c} \nabla J\left(\mu \right)=\lambda_{1}\nabla {||\mu ||}_{\text{TV}}+\lambda_{2}\nabla {||\Phi \mu ||}_{1}+2A^{*}\left(A\mu -y\right). \end{array}$$

As the *ℓ*_1_ norm and total variation term (16) is the sum of absolute values. The absolute value, however, is not a smooth function and as a result (19) is not well defined. In [24], Lustig et al. approximated the absolute value with a smooth function $\left|x\right|\approx \sqrt{x^{*}x+\xi }$, where *ξ* is a positive smoothing parameter. Then the gradient becomes $d\left|x\right|\approx (x/\sqrt{x^{*}x+\xi })$. We adopt this idea in our implementation. In particular, a smoothing factor *ξ* = 10^−15^ is used. 

## 4. Experimental Results

In this section, we present our experimental results. There are four sets of experiments. In the first two experiments, true CT images and simulated projections were used to study the performance of our algorithm under ideal and degraded conditions. The third and fourth experiments used real data collected using the Canadian Light Source (http://www.lightsource.ca/) and University of Saskatchewan facilities. In all cases, we investigated reconstructions from 20, 30, 40, 50, 60, 70, 80, 90, 100, 110, up to 120-view datasets extracted from the full dataset, respectively, representing different levels of data sampling. The study showed how the varying degree of sampling impacts the reconstruction. In each case, a uniformly spaced view angle data decimation scheme over 180° was used to obtain undersampled data. 

Reconstructions were quantitatively evaluated in terms of relative root mean square error (RRMSE), streak indicator (SI), and structural similarity (SSIM) index. The relative root mean square error (RRMSE) is defined as

(20) $$\begin{array}{c} \text{RRMSE}=\frac{{||y-y_{\text{ref}}||}_{2}}{{||y_{\text{ref}}||}_{2}}, \end{array}$$

where *y* is the reconstruction image by our proposed method and *y*_ref_ is the reference image. Since undersampling streak artifacts are an important feature in sparse-view CT image reconstruction, streaking level is also quantified by the streak indicator (SI) [25]. The streak indicator (SI) is defined as

(21) $$\begin{array}{c} \text{SI}=\text{TV}\left(y-y_{\text{ref}}\right). \end{array}$$

The lower the value of SI is, the less the streaking artifacts are present in the reconstructed image.

The structural similarity (SSIM) index is highly effective for measuring the structural similarity between two images [26]. Suppose *ρ* and *t* are local image patches taken from the same location of two images that are being compared. The local SSIM index measures three similarities of the image patches: the similarity of luminance *l*(*ρ*, *t*), the similarity of contrast *c*(*ρ*, *t*), and the similarity of structures *s*(*ρ*, *t*). Local SSIM is defined as

(22) S(ρ,t)=l(ρ,t)·c(ρ,t)·s(ρ,t)=(2μρμt+C1μp2+μt2+C1)(2σρσt+C2σp2+σt2+C2)(2σρt+C3σρσt+C3),

where *μ*_*ρ*_ and *μ*_*t*_ are local means, *σ*_*ρ*_ and *σ*_*t*_ are local standard deviations, and *σ*_*ρt*_ is cross-correlation after removing their means. *C*_1_, *C*_2_, and *C*_3_ are stabilizers. The SSIM score of the entire image is then computed by pooling the SSIM map, for example, simply averaging the SSIM map. Although in other papers, such as in [27], a metric name universal quality index (UQI) was used, SSIM is an improved version of the algorithm. Also, the correlation coefficient (CC) defined in [27] is also similar to SSIM. Hence, SSIM is highly effective for measuring image quality. Higher SSIM value indicates higher image quality.

**Figure 2:**
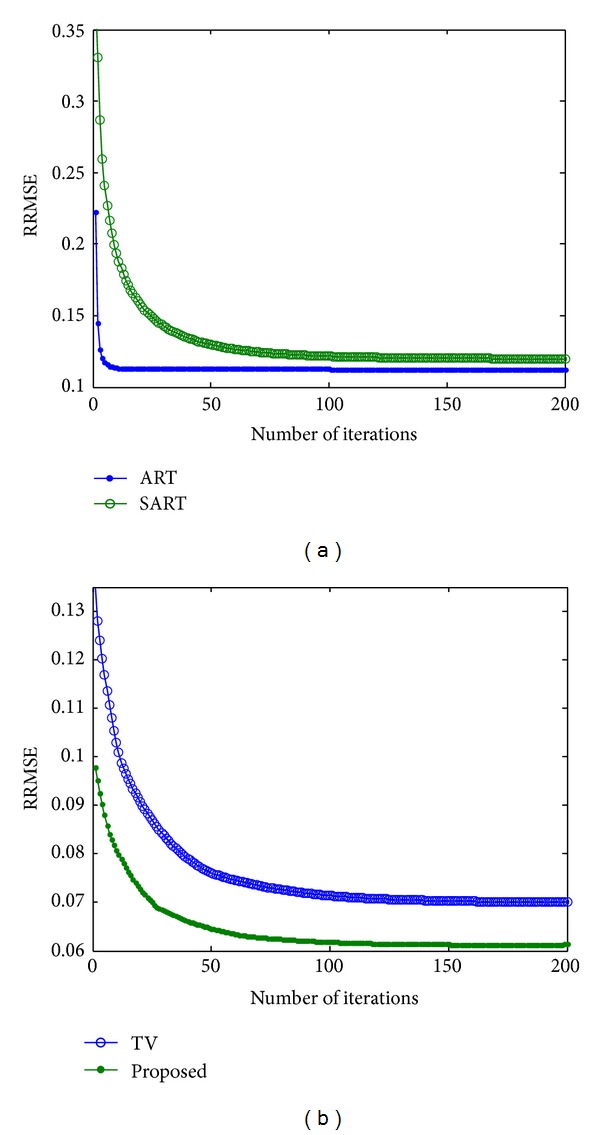
Analysis to find the optimum number of iterations for different methods: (a) ART and SART, (b) TV and the proposed scheme.

In order to find the optimum number of iteration, we have conducted another experiment using simulated phantom. The results are shown in Figure 2. It can be seen from Figure 2(a) that the RRMSE of ART becomes almost unchanged after 30 iterations. Hence, 30 is used as the optimum number of iterations for ART for all experiments. Similarly, the optimum number of iterations for SART, TV, and the proposed method is also estimated, and 150 is used for them. To verify the number of iterations, the experiments were repeated on noisy phantom and real data, and the results were found to be consistent with that of Figure 2.

**Figure 3:**
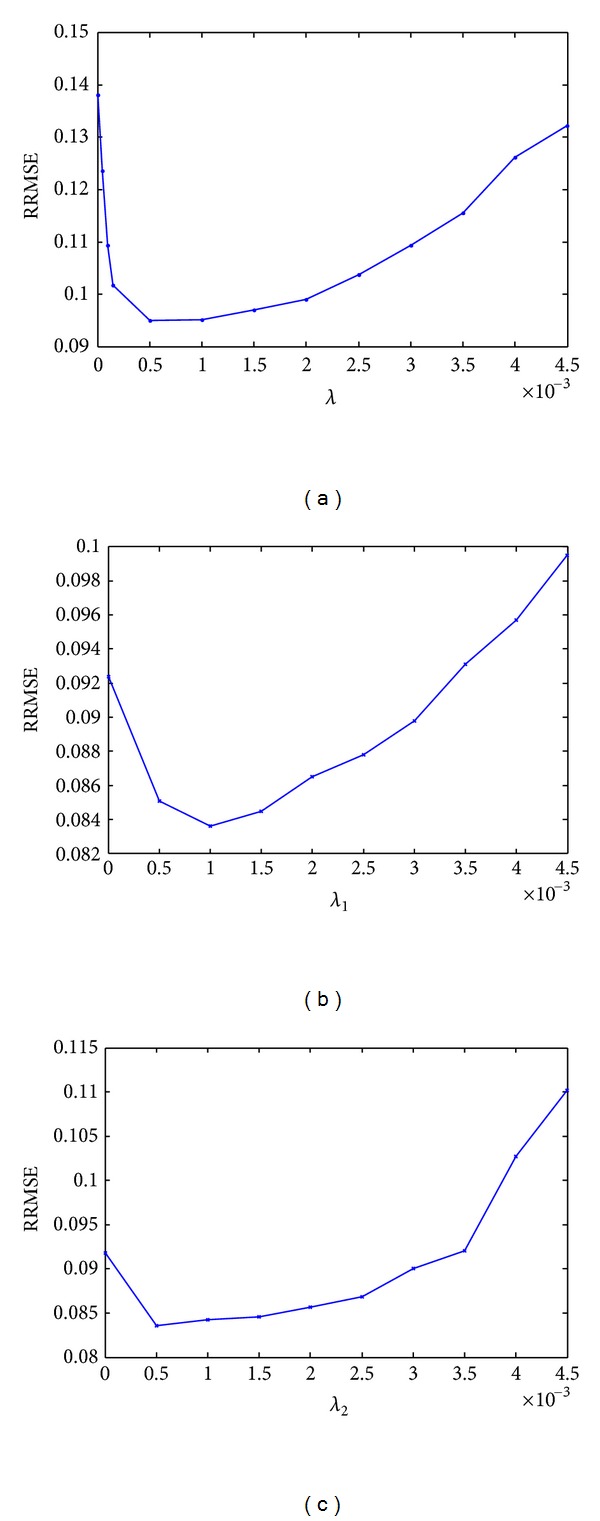
Analysis to find the optimum regularization parameters (for rat dataset): (a) *λ* in TV method; (b) *λ*_1_ when *λ*_2_ = 0.0005 for the proposed method; (c) *λ*_2_ when *λ*_1_ = 0.001 for the proposed method.

Moreover, the reconstruction accuracy depends on the selection of optimum regularization parameters for both TV method and the proposed method. We have used a real dataset (such as rat dataset as described later in Section 4.3) using 50 projections as an example to show the methodology of determining the optimal parameters. For TV method, the reconstruction error is plotted against *λ* (15), as shown in Figure 3(a). The lowest reconstruction error is obtained when *λ* is between 0.0005 and 0.001. In this study, we have selected *λ* = 0.0005. The optimal *λ* for all datasets is shown in Table 1. 

For the proposed algorithm, there are two parameters. We alternately plotted the reconstruction error against one parameter keeping the other fixed. We started by setting *λ*_2_ = 0.0005.  Figure 3(b) shows that the lowest reconstruction error is obtained when *λ*_1_ is 0.001. Then we set *λ*_1_ to 0.001 and searched the optimal value for *λ*_2_ that gives the lowest error, as shown in Figure 3(c). Thus, we used this recurring process to determine the optimum values of *λ*_1_ and *λ*_2_. Similar search was conducted for all dataset. The optimal values of these parameters are shown in Table 1. The full-view FBP reconstruction image was used as the reference.

### 4.1. Experiment Results Using Phantom

**Figure 4:**
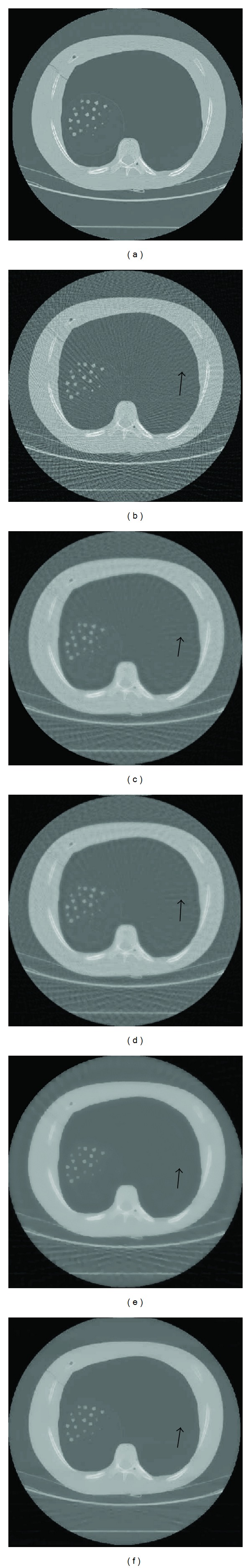
The reconstruction results of the nodule phantom using 50 projections. (a) The ground truth image, (b) the result obtained using FBP algorithm, (c) the ART algorithm, (d) the SART algorithm, (e) the TV algorithm, and (f) the proposed CS algorithm.

The first experiment was performed using nodule phantom image and simulated projection without any noise purposely added. This data is provided free of charge by the National Cancer Institute (NCI) [28]. We used one typical cross-section of CT slice as a sample set. We suppose that it is the desired CT image and each pixel value presents an attenuation coefficient. The sample image was 512 × 512. Simulated projections were obtained by computing the line integrals across the image with different views uniformly distributed over 180°. The reconstructed images using 50 projections are shown in Figure 4. As can be noticed from Figure 4(b), the conventional FBP algorithm is not able to reconstruct diagnostically satisfactory image with such few projections and strong streaking artifacts are present. Although streaking artifacts are reduced in ART and SART reconstructions, we can still see them in smooth regions, as indicated by black arrows in the figure. In contrast, even with fewer projections, both the TV method and the proposed algorithm can capture most of the structures, leading to visually much better results.

**Figure 5:**
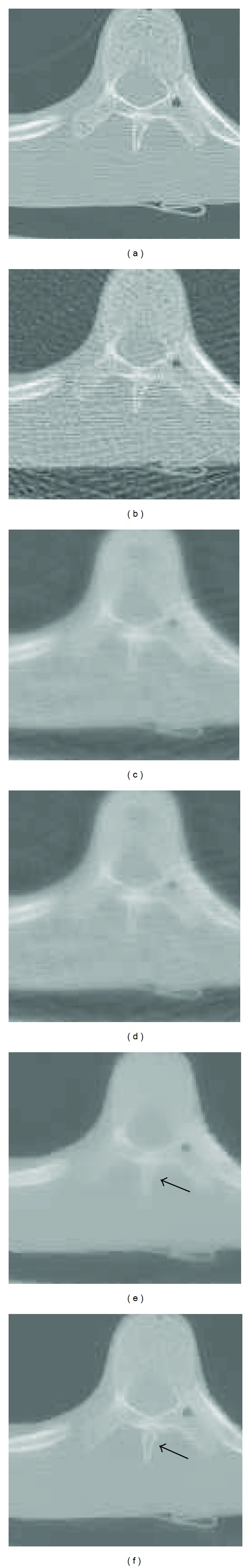
A detailed section of Figure 4: (a) ground truth, (b) FBP method, (c) ART method, (d) SART method, (e) TV method, and (f) the proposed method.

However, we can still see some residual streak artifacts in the TV reconstruction. The image reconstructed from our proposed method shows the least level of streaking artifacts. One possible reason for that is, in wavelet domain, the noise is uniformly spread throughout the coefficients while mostly the image information is concentrated in the few largest coefficients [29, 30]. Hence noise is of potentially small values in wavelet domain. As (18) tries to minimize the *ℓ*_1_ norm of wavelet coefficients, small values corresponding to noise and artifacts are also suppressed, leading to better reconstruction. Besides, all TV-based methods tend to remove small structure and degrade the image resolution and image quality. But compared to TV method, the proposed method has a slight advantage in preserving edges. To see it clearly, an expanded region is shown in Figure 5. We can see from the figure that both TV method and the proposed method can further remove the streaking artifacts that are presented in ART and SART reconstructions. But the fine structures get blurred as TV method suppresses the gradient of the image. As indicated by the black arrows, the low contrast edges are better reconstructed by our proposed method. To quantify the results, we also show the RRSME, SI, and SSIM values of the reconstructed images in Table 2. Clearly, the result from our method has lower error level, less streak artifacts, and higher structural similarity.

### 4.2. Experiment Results Using Phantom (with Noise)

**Figure 6:**
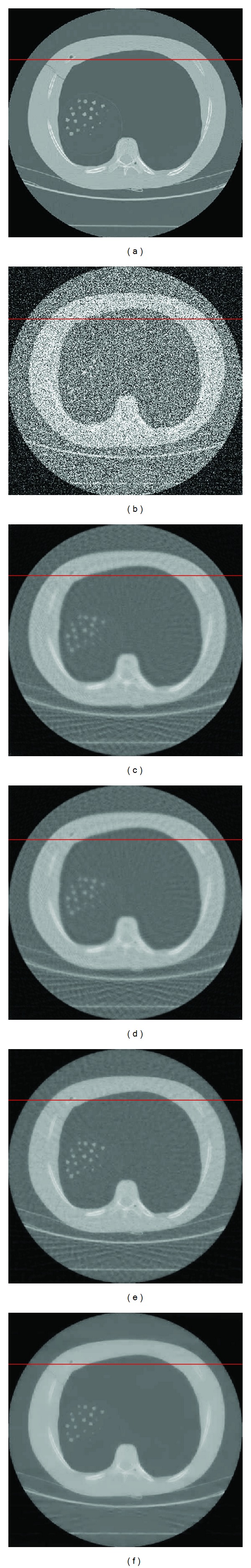
Simulated reconstruction of noisy phantom from 50 noisy projections over 180°: (a) the true image, (b) FBP, (c) ART, (d) SART, (e) TV, and (f) the proposed method.

**Figure 7:**
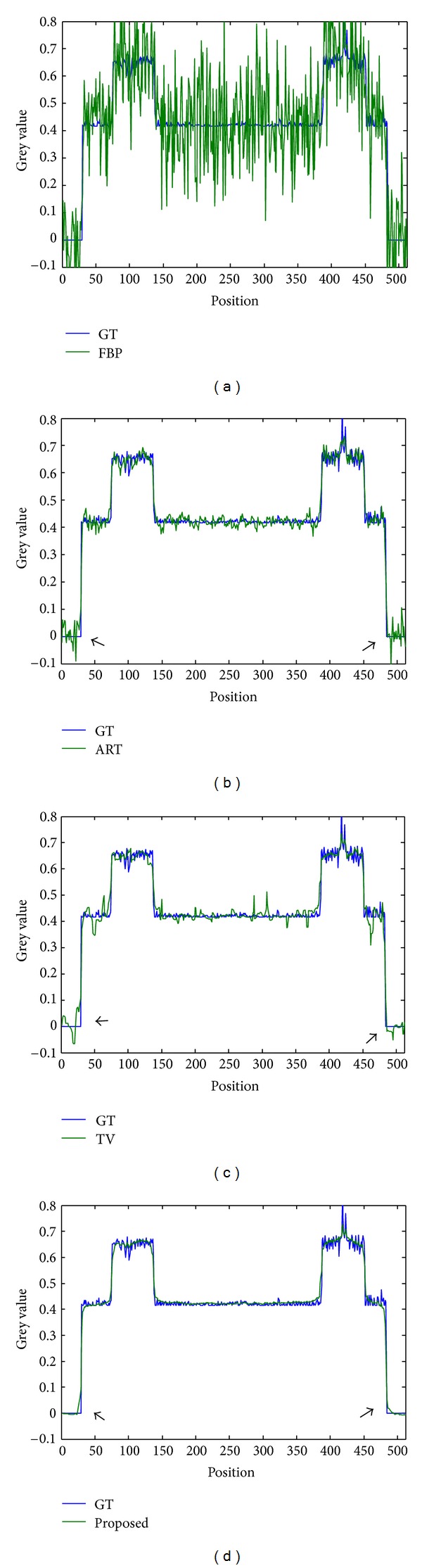
Pixel-intensity profiles of reconstructed images compared with ground truth (GT): (a) FBP, (b) ART, (c) TV, and (d) the proposed method.

The second experiment was performed using noisy simulated data. Additive Gaussian white noise *e* of relative magnitude ||*e*||_2_/||*Aμ*_true_||_2_ = 0.05 was purposely added to the sinograms. The results are displayed in Figure 6. To better compare the TV method and our proposed method, we also show horizontal line intensity profile going through the red line of Figure 6. The line intensity profiles are shown in Figure 7. Compared to FBP, the ART is more robust to noise and thus has greatly suppressed the streaking artifacts. SART produces similar results (not shown in Figure 7). But there are high frequency vibrations around the edges, as indicated by black arrows. The vibration is caused by limited view and added noise. The vibration is eliminated in terms of frequency and amplitude in the TV reconstruction. In contrast, the intensity profile of reconstructed image by the proposed method shows a rather smoothed curve in nonedge regions and is also much closer to the ground truth profile near the edges, demonstrating its ability to produce better edges. The results are summarized in Table 3. It was evident that our algorithm showed strong robustness against noise.

### 4.3. Experiment Results Using Real Dataset

**Figure 8:**
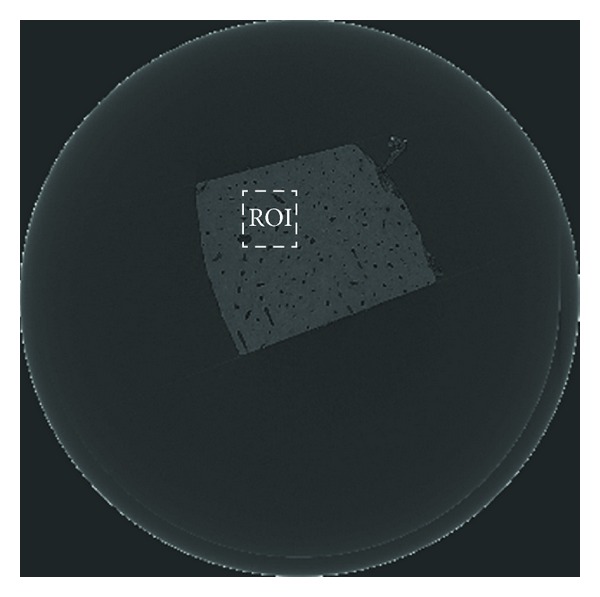
The FBP reconstruction of the complete dataset. The image has a large smooth region, so to better demonstrate the details, a region of interest (ROI) is selected.

 In the third and fourth experiments, we used real data collected from the Canadian Light Source facility and from a desktop Bruker SkyScan 1172 Micro-CT system with two datasets: human femoral cortical bone and the hindpaw of a normal Wistar rat. For the human bone, micro-CT scanning was performed at the BioMedical Imaging and Therapy Bending Magnet Beamline (BMIT-BM; 05B1-1). Projections were collected with a Hamamatsu C9300 (Hamamatsu Photonics, Hamamatsu, Japan) CCD camera fitted with a beam monitor with a 10 *µ*m thick gadolinium oxysulfide scintillator. The sample was rotated through 180° at 0.1 degree steps, generating 1800 original projections. The image size is of 3780 × 3780 pixels. We have selected a region of interest (ROI) from this image to further demonstrate the advantage on the reconstructed images. The FBP reconstruction using 1800 projections is shown in Figure 8. The last dataset in this study was a micro-CT scan of an adult Wistar rat hindpaw. This scan was taken at 70 kVp with the Bruker SkyScan 1172 Micro-CT in Anatomy and Cell Biology at the University of Saskatchewan. The reconstructed pixel size was 26.6 *µ*m. In total, 900 projections were acquired over a rotation through 180° at 0.2 degree steps. 

**Figure 9:**
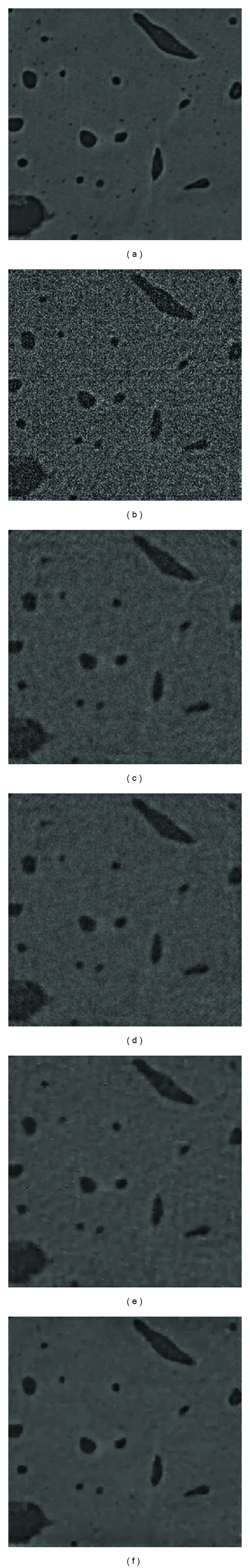
The ROI reconstructions of human bone. (a) The image reconstructed by FBP with 1800 projections, (b) the result obtained using FBP algorithm, (c) the ART algorithm, (d) the SART algorithm, (e) the TV algorithm, and (f) the proposed CS algorithm, all using 50-views.

The ROI reconstruction results restricted to 50 views for the human cortical bone image are shown in Figure 9. The gray tissue shown is the bone permeated with vascular canals, which appear darker in the image. Surrounding these larger canals some smaller objects can be seen. These are osteocyte lacunae, spaces within the bone where cells reside. The edges of the canals and lacunae are highlighted by propagation phase contrast halos. As expected the FBP reconstruction shows a greater amount of high spatial frequency noise over the entire area due to the limited sampling rate. The resolution is significantly diminished and many details of interest including the lacunae are lost. Image quality is lowered with strong and obvious streaking artifacts. 

In the ART and SART images, the streaking artifacts and noise are reduced, but residual artifacts can be seen and the noise is still pervasive. Besides this, they suffer from edge blurring artifacts and many low contrast structures are lost. The edges of the vascular canals are no longer able to be precisely distinguished, an important feature for characterizing their shape and size. The streaking artifacts in the TV reconstruction are less conspicuous than they are in FBP, ART, and SART, but we can clearly see some relatively low frequency patchy structures present in nonedge regions. In clinical practice, these patchy structures may mimic low contrast lesions and obscure the presence of small details. By comparison, our proposed method provides reconstruction of high fidelity, as presented in Figure 9(f). It is able to remove most of the streak artifacts without visible introduction of unwanted structures. For instance, the canals in the bone are much clearer in the image reconstructed by our proposed method than that in the image reconstructed by TV method, that their edges are much cleaner.

**Figure 10:**
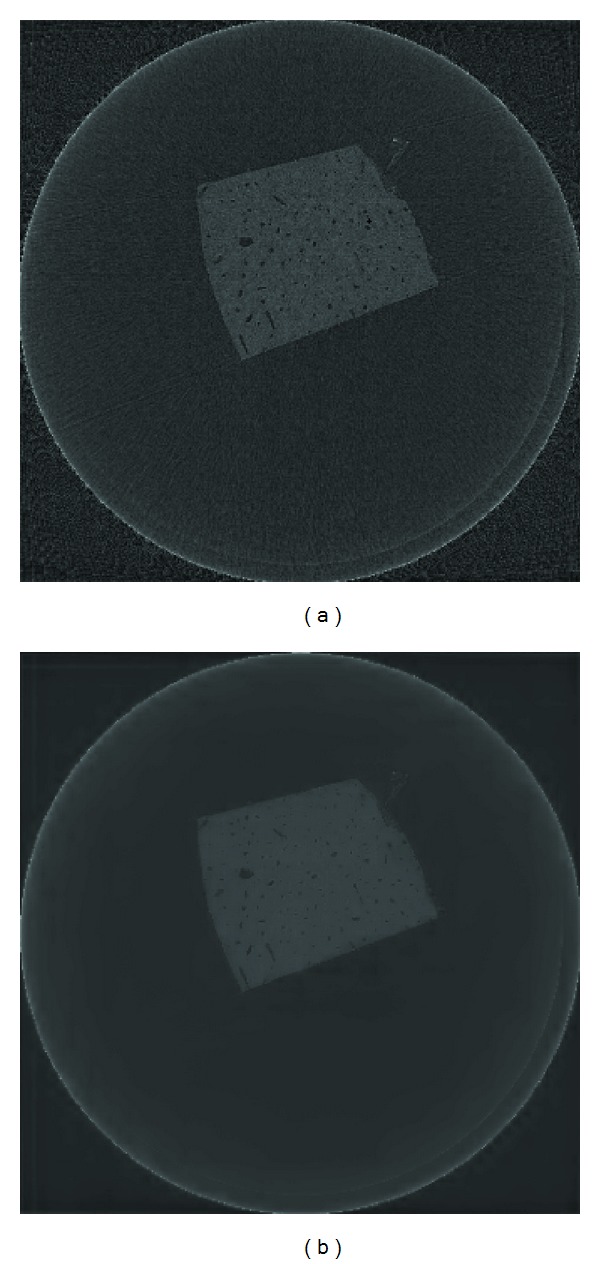
(a) The FBP reconstruction of human bone, (b) reconstruction using the proposed CS algorithm.

To further quantify the reconstruction accuracy and streaking artifacts, the RRMSEs, SIs, and SSIMs values of the given ROI by these methods are shown in Table 4. From the table, we can see that the RRMSE is well below 10% for both TV and our proposed method with the latter showing superior results. This result indicates that high reconstruction accuracy can be achieved using our proposed method. As well, from the SI value and visual observation of Figure 9, one may conclude that the proposed algorithm is capable of suppressing streaking artifacts and noise, leading to an image of acceptable quality at lower number of views. To highlight the ability of our proposed method to suppress streaking artifacts, the whole reconstructions by FBP and our proposed method are also shown in Figure 10. It is clearly seen from the figure that the streaking artifacts in FBP are greatly suppressed by our proposed algorithm.

**Figure 11:**
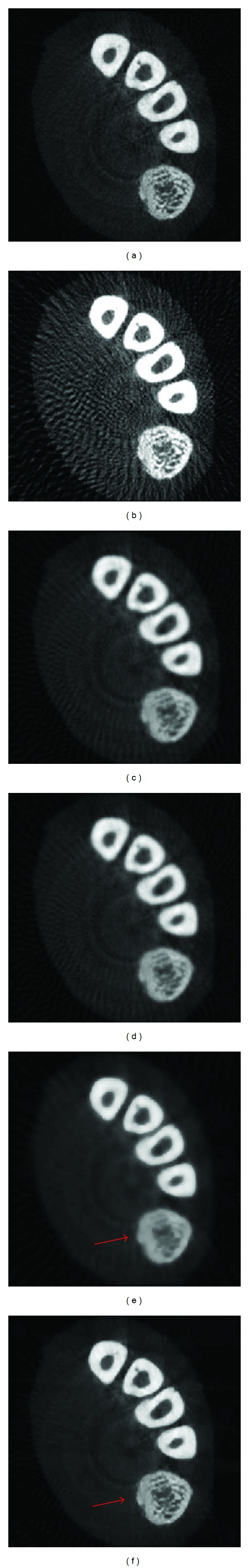
Reconstruction results of the hindpaw image of the adult rat. (a) FBP reconstruction using 900 projections, (b) FBP algorithm with 50 projections, (c) ART algorithm, (d) SART algorithm, (e) TV algorithm, and (f) the proposed CS algorithm, all using 50 views.

Now let us look at the adult Wistar rat hindpaw image. This image shows a transverse slice through the bones of the paw, with the bottom bone showing trabecular bone and the other four bones showing cortical bone and marrow cavities. The experimental results of the rat are displayed in Figure 11. Image quality is greatly degraded by obvious streaking artifacts in FBP reconstruction due to its inability to handle incomplete data. These artifacts are not efficiently removed by either ART or SART algorithms. By comparison, images reconstructed by the TV method and proposed method appear to have higher visual image quality, indicating that TV-based methods are superior to these methods.

Although the TV method can suppress the noise and streak artifacts considerably, it is still a great challenge to reconstruct the trabecular bone, the fine structure in the bottom right-hand corner of the image as indicated by the red arrows in Figure 11(e), because of the nature of total variation regularization. By the introduction of the wavelet transform in image reconstruction procedure, our proposed method minimizes noise and streaking artifacts both in the discrete gradient domain and wavelet domains, delivering better results than previous efforts without creating unwanted smoothing effects. Our method leads to a better reconstruction with higher spatial resolution.

**Figure 12:**
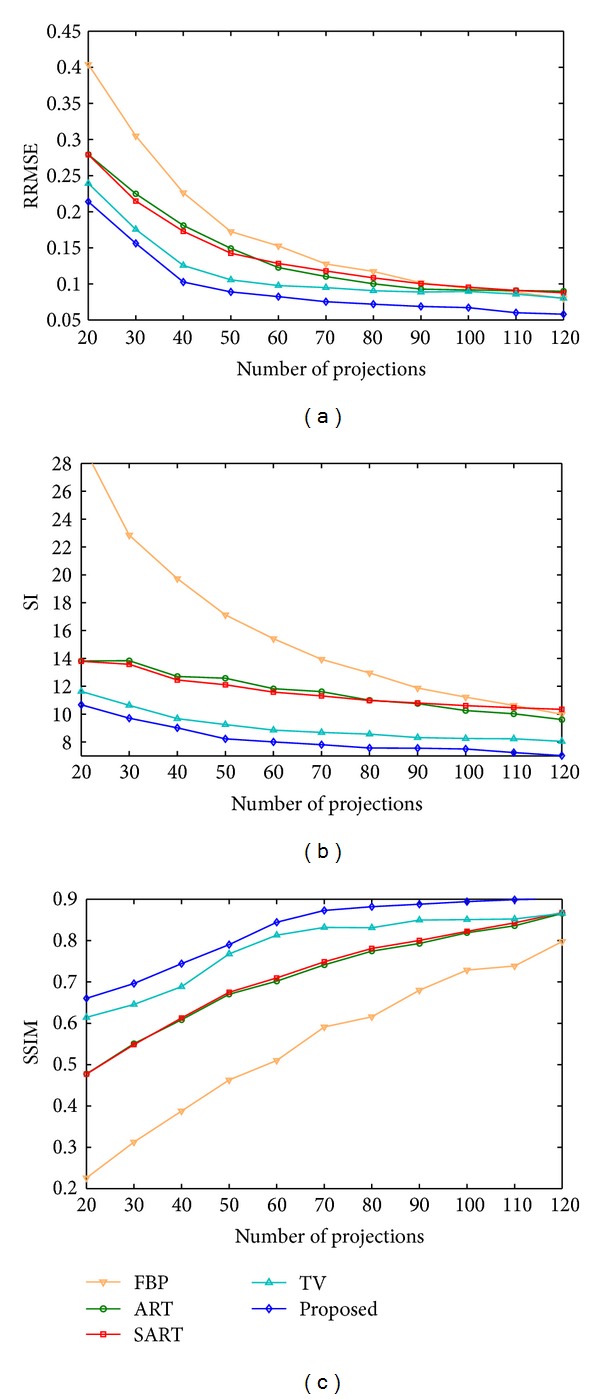
Plots of relative root mean square error (RRMSE), streak indicator (SI), and structural similarity (SSIM) for rat dataset.

For a comprehensive comparison, the RRMSEs, SIs, and SSIMs of the reconstructed images are also plotted against the number of projections in Figure 12. The shape of the curves shows the effectiveness of the corresponding reconstruction method in sparse-view regime. It also indicates that RRMSEs and SIs of reconstructions by our proposed method in all cases are lower than those of other methods while the SSIMs are higher than those of other methods. The results of this test confirm that our proposed method outperforms the TV method in maintaining the balance between noise suppression and spatial resolution preservation.

**Figure 13:**
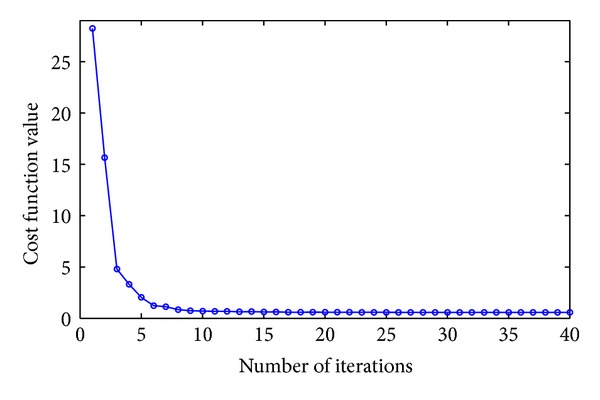
Convergence curve (cost function values versus number of iterations) for the proposed method applied to phantom dataset.

The convergence speed of an algorithm is a crucial factor for all iterative methods in clinical practice. To investigate the convergence speed of the proposed method, the plot of cost function value *J*(*μ*) in (18) against the number of iterations for the phantom dataset (without noise) is shown in Figure 13 with 30 views selected for demonstration. It shows that the curve decreases dramatically within 5 iterations, indicating the high convergence speed of our proposed method. 

## 5. Conclusion

In this work, we have investigated a novel compressed sensing-based algorithm for sparse-view CT image reconstruction, in which wavelet transform is used in the reconstruction procedure. Results show that the proposed method is able to suppress streak artifacts and noise caused by incomplete and noisy projection data without visible oversmoothing of fine structure details in the images. The proposed CS-based algorithm has potential to reduce the dose in clinical computed tomography imaging techniques.

## Figures and Tables

**Algorithm 1:**
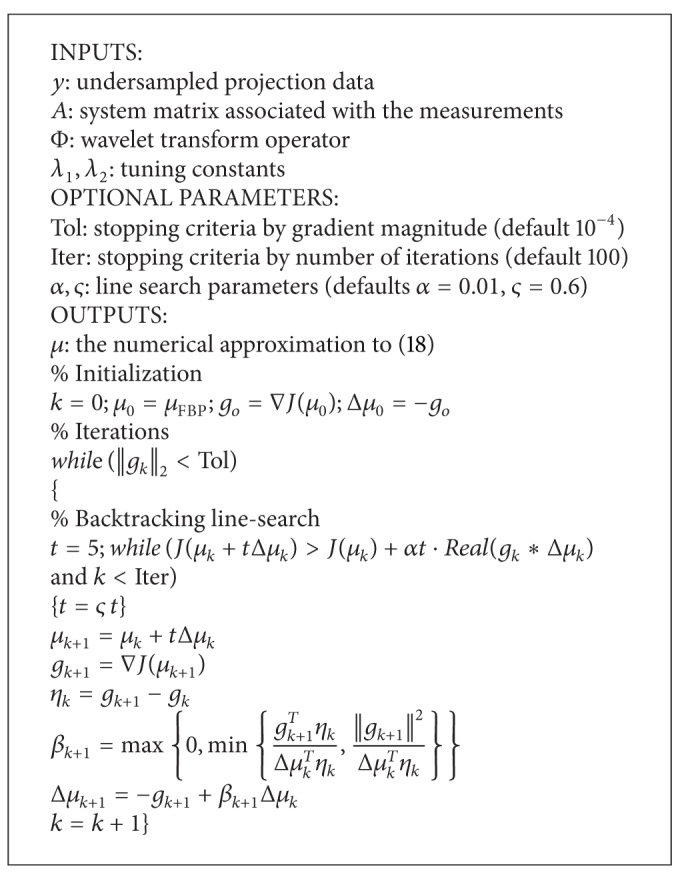
Iterative algorithm for *ℓ*_1_ minimization.

**Table 1:** Optimum parameter selections for each dataset.

Data	TV algorithm	Proposed algorithm	
	*λ*	*λ*_1_	*λ*_2_
Phantom without noise	0.0005	0.0005	0.0005
Phantom with noise	0.0015	0.001	0.0006
Human bone	0.001	0.001	0.001
Rat	0.0005	0.001	0.0005

**Table 2:** Reconstruction results using phantom image.

Reconstruction methods	RRMSE	SI	SSIM
FBP	0.1282	44.9556	0.6110
ART [17]	0.1120	25.5737	0.7681
SART [18]	0.1198	25.0023	0.7663
TV [12]	0.0715	20.0115	0.8716
Proposed method	0.0609	18.0646	0.9310

**Table 3:** Reconstruction results using phantom image (with noise).

Methods	RRMSE	SI	SSIM
FBP	0.2908	127.4656	0.3284
ART [17]	0.1197	28.7409	0.7260
SART [18]	0.1324	28.0063	0.7344
TV [12]	0.0891	24.1023	0.7693
Proposed method	0.0687	21.2074	0.8967

**Table 4:** Reconstruction results using real dataset.

Reconstruction methods	RRMSE	SI	SSIM
FBP	0.5102	97.325	0.3040
ART [17]	0.1525	22.9236	0.6893
SART [18]	0.1412	20.0544	0.6955
TV [12]	0.0783	6.7528	0.7983
Proposed method	0.0557	4.1120	0.8642
